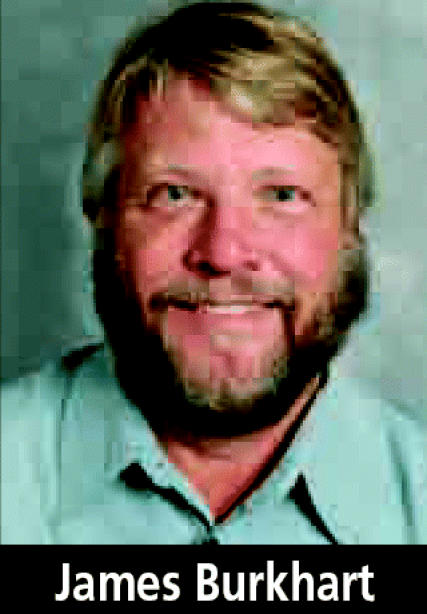# Farewell and Best Wishes

**Published:** 2007-01

**Authors:** James G. Burkhart

**Affiliations:** Acting Editor-in-Chief, *EHP*, National Institute of Environmental Health Sciences, National Institutes of Health, Department of Health and Human Services, E-mail: burkhart@niehs.nih.gov

With publication of this issue, I have reached a long-planned retirement from the NIEHS and *Environmental Health Perspectives* (*EHP*). The mission of *EHP* is to serve as a forum for the discussion of the interrelationships between the environment and human health by publishing in a balanced and objective manner the best peer-reviewed research and most current and credible news of the field. I feel privileged to have been a part of this effort. From the time I began as science editor of *EHP* in March 2002, my goal was to engage as directly as possible with the environmental science community so that *EHP* could best serve as a focal point for the dissemination of relevant research. Through the combined dedication and efforts of the *EHP* staff and editorial boards, the journal has progressed in both science and outreach. Our impact factor now places *EHP* at the top of two categories of science journals: environmental science and environmental, occupational, and public health. We are privileged to be part of a changing view of environmental health science, one that recognizes that improved environmental health requires creative integration across previously classically defined disciplines: environment, exposures, and individual/population health cannot be separated from genetic and societal contexts. The articles published in *EHP* over the last few years provide many examples of this understanding, and I hope that your future contributions to the journal will continue to reflect these ideas.

Believing that credible science information is a powerful means to improved health, I have been particularly proud to be a part of efforts to facilitate communication of environmental health sciences in two areas: to and within developing countries and to high school students. *EHP* ’s programs to disseminate environmental science worldwide, including our policy of open access, have provided much-needed access to vital information at a time of critical global development. Similarly, our student edition program engages the next generation of scientists in the compelling world of environmental health at a critical time in their academic development. The response by the worldwide science and education communities to these programs has been terrific. I hope that these unique efforts continue to provide a successful model for how research journals can serve to inform, educate, and improve the lives of people around the world.

Concurrent with my retirement, a search for a new editor-in-chief is being conducted. I invite you to become involved by bringing forth highly qualified candidates dedicated to environmental health and the future of *EHP*. In the meantime, Kenneth Korach, director of the Environmental Diseases and Medicine Program of the NIEHS, will serve as interim editor-in-chief. I will assist with various aspects of transition before beginning an eagerly anticipated extended road adventure, after which I will explore new avenues for involvement in global environmental health issues.

The passion and engagement of *EHP* ’s readers, combined with the excitement and dedication of our staff, have made the years of my tenure here the best for me to remember. On behalf of all the staff of *EHP*, I thank you for your tenacious support. Its impact on our morale and feelings about what we have tried to accomplish cannot be overstated. I leave with the deep conviction that the environmental health community is making a real difference in global health and that *EHP* will continue to be at the vanguard of these efforts. My best wishes for the coming year and all the ones to follow.

## Figures and Tables

**Figure:**